# Prevalence of Multi-Resistant Microorganisms and Antibiotic Stewardship among Hospitalized Patients Living in Residential Care Homes in Spain: A Cross-Sectional Study

**DOI:** 10.3390/antibiotics9060324

**Published:** 2020-06-13

**Authors:** Mario Rivera-Izquierdo, Alberto Benavente-Fernández, Jairo López-Gómez, Antonio Jesús Láinez-Ramos-Bossini, Marta Rodríguez-Camacho, María del Carmen Valero-Ubierna, Luis Miguel Martín-delosReyes, Eladio Jiménez-Mejías, Elena Moreno-Roldán, Pablo Lardelli-Claret, Virginia Martínez-Ruiz

**Affiliations:** 1 Service of Preventive Medicine and Public Health, Hospital Universitario San Cecilio, 18016 Granada, Spain; mariac.valero.sspa@juntadeandalucia.es; 2 Department of Preventive Medicine and Public Health, University of Granada, 18016 Granada, Spain; luismiguelmr@ugr.es (L.M.M.-d.); eladiojimenez@ugr.es (E.J.-M.); elmorol@ugr.es (E.M.-R.); lardelli@ugr.es (P.L.-C.); virmruiz@ugr.es (V.M.-R.); 3 Instituto de Investigación Biosanitaria de Granada, ibs.GRANADA, 18012 Granada, Spain; 4 Programme in Clinical Medicine and Public Health, University of Granada, 18016 Granada, Spain; ajbossini@ugr.es; 5 Service of Internal Medicine, Hospital Universitario San Cecilio, 18016 Granada, Spain; alberto.benavente.sspa@juntadeandalucia.es (A.B.-F.); jairo.lopez.sspa@juntadeandalucia.es (J.L.-G.); 6 Service of Neurology, Hospital Universitario San Cecilio, 18016 Granada, Spain; marta.rodriguez.camacho.sspa@juntadeandalucia.es; 7 Consorcio de Investigación Biomédica en Red (CIBER) de Epidemiología y Salud Pública de España, CIBERESP, 28029 Madrid, Spain

**Keywords:** multidrug resistance, care homes, nursing homes, antibiotic stewardship, hospitalization, elderly, internal medicine, Spain

## Abstract

Antimicrobial resistance is a growing global health problem. Patients living in care homes are a vulnerable high-risk population colonized by multidrug-resistant organisms (MDRO). We identified a case series of 116 residents of care homes from a cohort of 540 consecutive patients admitted to the internal medicine service of our hospital. We performed early diagnostic tests of MDRO through anal exudates in our sample. The prevalence of MDRO colonization was 34.5% of residents and 70% of them had not been previously identified in the clinical records. Previous hospitalizations and in-hospital antibiotic administration were significantly associated with the presence of MDRO. Our results emphasize the need to consider care homes in the planning of regional and national infection control measures and for implementing surveillance systems that monitor the spread of antimicrobial resistance in Spain. Systematic early testing upon admission to hospital services with a high prevalence of patients with MDRO colonization (e.g., internal medicine) could contribute to the adoption of adequate prevention measures. Specific educational programs for care home staff should also be implemented to address this increasing problem.

## 1. Introduction

Antimicrobial drug resistance (AMDR) is a major public health problem of global concern [1]. The inappropriate use of antimicrobial drugs has become one of the main causes of increased AMDR worldwide [2]. Therefore, current strategies to combat AMDR are primarily focused on antimicrobial stewardship [3,4,5]. Moreover, according to the One Health perspective, AMDR can occur for different reasons, including human, animal or environmental factors [6,7]. Therefore, a collaborative effort of the different health science disciplines seems necessary to address this major issue. 

In Spain, nosocomial AMDR continues to increase in frequency and is associated with significant morbidity and mortality [8]. In March 2018, the Spanish Society of Infectious Diseases and Clinical Microbiology (SEIMC) conducted a study to determine the mortality of multidrug-resistant (MR) infections. According to the results of this study, 35,400 annual deaths due to multidrug-resistant organisms (MDRO) were estimated in our country [9]. As documented in the European Antimicrobial Resistance (EARS-net) surveillance study [10], AMDR has increased in Spain for almost every group of antibiotics in recent years. In addition, antimicrobial stewardship is well above the recommendations [11], especially regarding the Critically Important Antimicrobials defined by the World Health Organization [12]. This may partially explain, for example, the twofold increase in MR *Klebsiella pneumoniae* or the ≈ 33% increase in MR *Escherichia coli* in our environment [11].

Nevertheless, AMDR does not affect all populations equally. The role of care homes has been increasingly recognized as a key factor in the transmission and spread of AMDR through the healthcare network [13]. Although the daily probability of microbial transmission in nursing homes is significantly lower than in hospitals, the transmission of MDRO may be more effective given the increased length of stay of residents. A recent systematic review found a high frequency of AMDR in care homes [14]. In Spain, the transmission of methicillin-resistant *Staphylococcus aureus* (MRSA) and a high rate of quinolone-resistant MRSA have been reported in community care homes [15]. Although the aforementioned review did not show conclusive results regarding the usefulness of strategies to reduce AMR in care homes [14], some evidence shows promising results. For instance, a recent antimicrobial stewardship intervention for older people in nursing homes conducted in North Carolina significantly reduced urine culture positive MDRO rates [16]. Based on these facts, it seems clear that nursing homes, care homes and long-stay residences should be considered in addition to hospitals when planning regional and national infection control strategies to reduce AMDR. 

The recent 2019 coronavirus disease (COVID-19) pandemic has brought to light the precarious position of Spanish care homes [17], where older and vulnerable patients are concentrated in enclosed spaces. According to 2019 data, the number of care homes in Spain was 5417, of which 3844 (71%) were privately run, housing a total of 271,696 residents [18]. The lack of political and social consideration as well as the urgent need for adequate human and technical resources have been magnified during this pandemic, highlighting that competent local administrations and governments should provide care homes with adequate safety equipment and ensure the appropriate training of their staff [17].

This issue can be extrapolated to AMDR transmission. Following the One Health concept, care home residents admitted to hospitals represent a potential opportunity for the early detection and reduction in AMDR transmission based on a multidisciplinary approach. Currently, the most common procedure consists of analyzing AMDR in the anal exudates of patients who are discharged from hospital to a care home [19], especially in services such as internal medicine, where the prevalence of patients living in residential care homes is likely to be high. We hypothesized that the identification of anal exudates from early hospital admission might optimize the adoption of preventive control measures. However, no specific data on AMDR prevalence in Spanish care home residents is available. Therefore, the aim of this study was to evaluate the presence of MDROs, previous antibiotic stewardship and the impact of an early diagnostic intervention through anal exudates among patients living in residential care homes admitted to the internal medicine service in a tertiary hospital in Granada (Spain).

## 2. Methods 

### 2.1. Study Setting and Design

A cross-sectional case series study was conducted in our institution (Hospital Universitario San Cecilio, Granada, Spain) from 1 January to 29 February, 2020. A total of 540 consecutive patients were admitted to the internal medicine service during this period. Of them, 116 (21.5%) lived in care homes and, therefore, were eligible for our study. Inclusion criteria included living in care homes and voluntary acceptance to participate in the study or, in the case of cognitive deterioration, voluntary acceptance by a family member. Finally, all 116 patients were included and constituted our study sample.

### 2.2. Data Collection

Socio-demographic characteristics and patients’ history of previous diseases were collected through clinical records and interviews with patients or their relatives. On the first day of hospital admission, each patient was interviewed, and an anal exudate to detect MDRO colonization was performed in all cases. Information regarding patients’ care homes was collected through epidemiological regional databases, mainly the Andalusian Surveillance Epidemiological System (SVEA). In order to calculate mortality rates, data collection ended on 14 April, 2020, when all patients had completed the hospitalization process (i.e., discharge or death).

### 2.3. Analysis

A descriptive analysis was made using R statistical software through the R-Commander application (R Core Team, 2013, http://www.R-project.org/). Shapiro–Wilks tests were applied, and normality was confirmed for the analyzed variables. Differences between colonized and non-colonized patients living in care homes were assessed through T-tests (for quantitative variables) and chi-square tests (for qualitative variables).

### 2.4. Ethical Considerations

This study was performed in accordance with the ethical standards laid down in the 1964 Declaration of Helsinki and its later amendments. This study was approved by the Regional Ethics Committee (CEIM/CEI Provincial de Granada), code 0863-N-20. All patients were informed and voluntarily agreed to participate or, in case of cognitive deterioration, voluntary acceptance by a family member was obtained. The database was anonymized, and any variable including information that could lead to patient identification was removed for the analyses.

## 3. Results

The mean age of patients in our series was 83.5 years (SD: 12.6) and 69.0% were females. The in-hospital mortality rate was 20.7%. The prevalence of MDRO colonization was 34.5% (40 out of 116). Only 12 (30%) colonized patients were previously included in the databases of patients’ clinical records; therefore, 70% of MDRO-colonized residents were identified by systematic early diagnosis for the first time. Moreover, only four (10%) of the colonized patients had a history of infection caused by the same agent. 

Care homes of all colonized patients from our sample were identified. Only 50% of these care homes had been previously identified and were monitored in the available epidemiological databases. Therefore, half of the colonized patients were living in a care home where existing cases colonized with MDROs had not been previously identified and appropriate infection control measures had not been adopted in their epidemiological districts.

Table 1 shows the distribution of microorganisms in the 40 patients with positive anal exudates. The most prevalent bacteria were extended-spectrum beta-lactamase (ESLB) *Escherichia Coli* (40%), ESLB *Klebsiella pneumoniae* (32.5%), and ESLB OXA-48 *Klebsiella pneumoniae* (27.5%).

Table 2 shows the distribution of previous diseases, dependence for activities of daily living, and previous hospitalization characteristics of patients living in care homes, overall and stratified according to their MR status. A high prevalence of previous diseases was found, mainly hypertension (65.5%), cardiovascular disease (48.3%), neurological disorders (44.8%) and diabetes mellitus (37.9%). No patient living in care homes was independent for activities of daily living (37.9% partially dependent, 62.1% completely dependent). No differences were found between colonized and non-colonized patients. However, the number of hospitalizations during the last year (*p* = 0.021), hospitalization due to infection with a confirmed diagnosis (*p* = 0.018) and the number of hospitalization days during the last year (*p* = 0.002) were significantly higher in colonized patients. Of the 20 patients that received antibiotic treatment, 17 (85.0%) received more than one antibiotic and nine (45.0%) received carbapenems. The average number of treatment drugs at home (multi-drug use) was 7.8 (SD: 3.1).

## 4. Discussion

In this study, we carried out an early diagnostic intervention based on obtaining an anal exudate from patients living in a care home who were admitted to the internal medicine service in our hospital. The frequency of patients living in these facilities in the sample analyzed was 21.5%, which favors the hypothesis that the prevalence of this population in the internal medicine service is high. Anal exudate was the diagnostic test selected based on the previously reported prevalence of MDROs in our environment [8,9,10,11], since most of these pathogens can be identified through this technique. As a first exploratory study, we aimed to identify most of the MDROs, while avoiding an excessive number of annoying tests performed on patients. However, future studies should consider broadening the set of early diagnostic exams.

In our hospital, the early identification of MDROs on admission is only routinely implemented in the Intensive Care Unit (ICU), which has been widely reported to be a service with high rates of MDRO colonization [20,21]. 

Furthermore, approximately one third of patients presenting to hospital from care homes were colonized with MDROs. It seems evident that the proportion of patients colonized with MDROs overestimates the actual prevalence of MDRO colonization in care homes, since we selected a sample of patients that required hospitalization. In addition, most patients colonized with MDROs were unknown to the epidemiological surveillance system (70% in our sample), which implies that they would go unnoticed in the absence of early detection programs upon hospital admission. Because of this, they become a potential undetected source of transmission and spreading of MDROs both in their care homes and in hospitals. This fact encourages us to believe that early diagnosis based on routine identification of MDRO colonization might be an efficient strategy in this vulnerable high-risk population. Although anal exudates proved to be useful in our study, they might be insufficient for exhaustive and comprehensive detection of MRDOs. Therefore, nasopharyngeal exudates and surveillance strategies for high-risk groups (i.e., in residential care homes) should also be implemented.

Of particular concern was the prevalence of carbapenemase OXA-48-producing *Klebsiella pneumoniae*, which accounted for 20% of colonized patients. This germ has been reported to be an emergent pathogen in our environment, with a wide range of antibiotic resistance and high gene variability both in hospital samples [22,23] and wildlife [24] in Spain. In our clinical experience, ESLB *Klebsiella pneumoniae* tends to acquire the carbapenem resistance plasmid and, therefore, overuse of these antibiotics could be optimized to avoid resistance selection.

Nevertheless, it is very difficult to determine the original source of transmission in this population. The crucial role of care homes in the transmission of MDRO infections has been consistently reported [13,14,15,16]. A number of factors could explain this high transmission, e.g., healthcare personnel simultaneously working in several care homes, the high rate of hospitalization and antibiotic use in this vulnerable population, or a lack of resources and training in infection prevention measures, as previously suggested in Spain [17]. On the other hand, we found a higher frequency of previous hospitalizations in MDRO-colonized patients compared to non-colonized patients. Therefore, it is possible that patients living in care homes, who are more vulnerable due to advanced age and concomitant diseases, represent a population at particular risk for frequent hospitalizations. In such a case, previous hospitalizations, where antibiotics are often and widely prescribed, might be the source of contagion or selection of MR strains. According to the EPINE-EPSS Study (Prevalence of infections and use of antimicrobial drugs in Spanish hospitals), the prevalence of nosocomial infections was 7.7% in 2019, which has not significantly changed over the last decade [25]. We also found a high use of antibiotics in this population during hospitalization (30%); therefore, strategies aimed at decreasing antibiotic stewardship (i.e., reducing the high rate of carbapenem prescriptions) could help to improve these rates.

In any case, regardless of the source of transmission, the prevalence of MDRO colonization in patients living in care homes in Spain is high. Therefore, this population represents an opportunity for optimizing preventive infection measures and reducing the burden of MR transmission. Currently, predictive models are being developed to quantify future antimicrobial resistance in Spain and other countries [26], considering the threat of the human microbiome [27], food animals [28] and the overuse of antibiotics [29]. In Spain, antibiotic stewardship could be addressed both in hospitals and primary care services based on multidisciplinary programs involving different healthcare professionals, particularly pharmacists [30]. In fact, a prospective study with an actress visiting 220 pharmacies concluded that the training of pharmacists in dispensing antibiotics should be improved [31]. However, our results clearly indicate that specific programs and practices in care homes should also be developed. In Europe, interventions to optimize antibiotic prescription for older people in care homes showed effective results [32] given the high rate of stewardship [33]. Several programs that address preventive measures to avoid MDRO have previously proved to be cost-efficient in Spain [34,35].

A nationwide “Zero Resistance” program has been implemented by the Spanish Ministry of Health with the aim being to reduce the selection and dissemination of MR pathogens in the ICU [36]. We propose to broaden the control measures established by this program to other services with a particularly high frequency of care home residents, such as Internal Medicine. These measures could include the early identification of MR colonization through anal exudates, early infection control measures during hospitalization and effective communication with care homes.

Another cornerstone for future research lies in the identification of patient profiles at high risk of MR colonization. In our sample, all patients were dependent for activities of daily living and, therefore, they represent a high risk of transmission given their needs in hygiene assistance. A significant number of them had been hospitalized the previous year and received more than two antibiotics during the hospitalization process. In this preliminary study, we identified a profile of patients at risk of MR colonization, which could allow their early in-hospital detection and the subsequent prevention of MR transmission. Future studies will be required to corroborate the effectiveness of programs addressing these patients and determine their exact role in MR dissemination.

On the other hand, apart from in-hospital measures, education on preventive measures should be implemented for the staff working in care homes based on multidisciplinary approaches. However, such measures should be designed with caution since aggressive isolation measures, differential care or movement restriction could make colonized residents excessively uncomfortable and even have a negative impact on their health.

Therefore, it is necessary to consider care homes in the planning of regional and national infection control measures and in the implementation of surveillance systems that monitor the spread of antimicrobial resistance in Spain. To the best of our knowledge, this is the first study conducted in Spain that analyzed the prevalence of MR microorganisms in patients living in care homes hospitalized in non-ICU services based on early diagnostic testing performed upon hospital admission. 

### 4.1. General Recommendations for Avoiding Drug Resistance in Care Homes

Early identification of MDROs in patients living in care homes—as well as in other risk groups—could help reduce the burden of antibiotic resistance. Diagnostic tests might be performed at any opportunity (both at hospital admission and in care homes). Antibiotic stewardship is another key element for avoiding drug resistance. Therefore, antibiotic prescription, especially the rate of in-hospital carbapenem prescription, should be reasonably optimized. The identification of MDROs must be followed by preventive control measures, particularly adequate hand hygiene [2]. Accordingly, the universal adoption of alcohol-based hand rub should be improved through multimodal interventions. Compliance with other infection control methods such as strict environmental cleaning, effective sterilization of reusable medical equipment and the use of contact precautions when it is indicated must also be considered [37]. Finally, educational strategies on infection preventive measures should be strengthened in healthcare workers and implemented for all the staff working in residential care homes.

### 4.2. Limitations 

Our study presents several limitations. Firstly, it is based on a cross-sectional case series design with a limited sample size (*n* = 116), which prevents us from obtaining conclusive results. Secondly, we recommended an early identification diagnostic strategy exclusively in the internal medicine service, where we believed that a highest frequency of patients living in care homes would be found. However, this hypothesis should be verified, and the frequency of this population in other services should also be considered in future studies (i.e., orthopedics, neurology, psychiatry), in order to better optimize our strategy. We also performed our analysis during high-demand months (January and February). Thirdly, due to feasibility criteria and the high prevalence of MR enterobacteria in our environment, we only considered anal exudates in this study. Therefore, the prevalence of other highly frequent MR microorganisms such as MRSA (mainly identified through nasopharyngeal exudates) was not considered in our series.

### 4.3. Future Approaches and Research

As a public health problem, MR in care homes requires a multimodal approach based on (1) the early detection of patients at high risk of colonization both in hospitals and health centers; (2) the adequate use of antibiotics during hospitalization and in residential care homes; (3) colonization screening before discharge; (4) effective communication with care homes for the management of these patients without affecting their quality of life and care; (5) the optimization of the roles of Preventive Medicine, Public Health and Epidemiology services as the links between hospitals and care homes, and (6) the development and evaluation of educational programs for the personnel working in care homes.

Future research should focus on better knowledge regarding the prevalence of MR in patients living in care homes in other countries and contexts, as well as the main modifiable risk factors, antibiotic stewardship of this population both in the residences and during hospitalizations, and the implementation of educational programs to optimize reasonable control measures in care homes.

## Figures and Tables

**Table 1:** Identified pathogens in patients living in care homes with laboratory-confirmed multidrug-resistant organism colonization in anal exudate culture (*n* = 40).

MDRO	x (s), *n* (%)
*ESLB Escherichia coli*	16 (40.0%)
*ESLB Klebsiella pneumoniae*	13 (32.5%)
*ESLB, OXA-48 Klebsiella pneumoniae*	11 (27.5%)
*MR Acinetobacter baumanii*	4 (10.0%)
*MR Pseudomonas aeruginosa*	2 (5.0%)
*ESLB, OXA-48 Escherichia coli*	1 (2.5%)
MR *Stenotrophomonas maltophilia*	1 (2.5%)

Multidrug-resistant organism (MDRO); extended-spectrum beta-lactamase (ESLB); carbapenemase OXA-48 enzyme (OXA-48). In total, more than 40 bacteria were identified, since some patients were colonized by more than one MDRO.

**Table 2:** Socio-demographic characteristics and previous diseases of patients living in care homes admitted to the internal medicine service.

Variable	Total Sample (*n* = 116)	MR ^1^ (*n* = 40)	Non-MR ^1^ (*n* = 76)	*p*-Values ^2^
**Previous diseases**	N (%)	N (%)	N (%)	
Hypertension	76 (65.5)	28 (70.0)	48 (63.2)	0.713
Diabetes mellitus	44 (37.9)	20 (50.0)	24 (31.6)	0.432
Cardiovascular disease	56 (48.3)	28 (70.0)	28 (36.8)	0.089
Chronic pulmonary disease	20 (17.2)	8 (20.0)	12 (15.8)	0.775
Chronic renal disease	20 (17.2)	8 (20.0)	12 (15.8)	0.775
Psychiatric disorders	20 (17.2)	8 (20.0)	12 (15.8)	0.775
Neurological disorders	52 (44.8)	12 (30.0)	40 (52.6)	0.244
Cognitive deterioration	72 (62.1)	20 (50.0)	52 (68.4)	0.331
**Dependence for activities of daily living**				0.868
Independent	0 (0.0)	0 (0.0)	0 (0.0)	
Partially dependent	44 (37.9)	16 (40.0)	28 (36.8)	
Completely dependent	72 (62.1)	24 (60.0)	48 (63.2)	
**Previous hospitalizations**				
Hospitalization DLY	52 (44.8)	32 (80.0)	20 (26.4)	0.021
Hospitalization for infectious diagnosis DLY	28 (24.1)	20 (50.0)	8 (10.5)	0.018
Antibiotic administration in hospitalization DLY	20 (17.2)	12 (30.0)	8 (10.5)	0.087
Hospitalization during the last 5 years	68 (58.2)	32 (80.0)	36 (47.4)	0.189
	Mean (SD)	Mean (SD)	Mean (SD)	
Hospitalization days DLY	7.03 (11.9)	15.8 (15.8)	2.42 (5.5)	0.002
Number of domiciliary treatment drugs (SD)	7.8 (3.1)	7.7 (3.1)	7.9 (3.2)	0.875

During the last year (DLY). Patients with laboratory confirmed colonization with MDRO in anal exudate culture (^1^MR). Patients with negative MDRO culture in anal exudate (Non-MR). ^2^ *p*-value for the differences between MR and Non-MR groups (T-tests for quantitative variables and chi-square tests for qualitative variables).
